# Supplementary material for: Transverse spin forces and non-equilibrium particle dynamics in a circularly polarized vacuum optical trap
Source: Nat Commun. 2018 Dec 21;9:5453. doi: 10.1038/s41467-018-07866-8 (PMC6303319; doi:10.1038/s41467-018-07866-8)
Supplement: Supplementary file 1 — Supplementary Information [file 41467_2018_7866_MOESM1_ESM.pdf]

Transverse spin forces and non-equilibrium particle dynamics in a circularly polarized vacuum optical trap : Supplementary Information

V. Svak, O. Brzobohatý, M.Šiler, P. Jákl, J. Kaňka, P. Zemánek, and S. H. Simpson*

The Czech Academy of Sciences, Institute of Scientific Instruments,

*Královopolská 147, 612 64 Brno, Czech Republic**

* simpson@isibrno.cz

CONTENTS

Supplementary Note 1. Aspects Of Optical Momentum 2
A. Momentum decompositions and coupling to matter 3
B. Transverse Optical Momentum In Counter-Propagating Circularly Polarized Gaussian Beams 4

Supplementary Note 2. Optical Forces 7
A. Small particle approximation 7
B. Mie particles 9

Supplementary Note 3. Langevin Equation Of Motion 11
A. Linear Regime, Characteristic Frequencies 13
B. Power Spectrum 15
C. Variance And Correlations 18
D. Parametric Survey 20

Supplementary Note 4. Orbital Motion 22
A. Equilibrium Conditions 22
B. Linear Stability 23
C. Orbits Fluctuations 26

Supplementary Note 5. Data Fitting 28

References 30

Supplementary Note 1. ASPECTS OF OPTICAL MOMENTUM

In the following section we briefly review the various ways in which optical momentum can be decomposed into distinct components, and how those components couple to matter. We provide a qualitative justification for our particular choice. Next, we show how the elected spin and orbital components of the momentum are distributed within counter-propagating, circularly polarized beams.

A. Momentum decompositions and coupling to matter

In this article, we claim that the observation of a transverse spin force is evidence of transverse spin momentum. This association is not trivial: optical momentum can be decomposed in various ways, and the ways in which these components couple to matter is open to interpretation. Furthermore, momentum is a point-wise property of the field, while forces are integral quantities. We note that the connection between optical force and momentum is a fundamental issue, effecting the interpretation of all such experiments. The relationship between optical torque and spin angular momentum is analogous. Circularly polarized light was first observed to rotate objects by Beth in 1936 [1], yet the interpretation of this experiment continues to be debated [2].

Under the conditions of our experiment (e.g. quasi-paraxial light beams incident on spherical, dielectric particles in vacuum), the possibilities are restricted. Below, we provide a rationale for the interpretation of our experimental results.

The Poynting momentum can be decomposed into distinct components in various ways. In continuous media careful consideration of this issue underpins discussions of the Abrham-Minkowski dilemma e.g. [3, 4]. In vacuum, decompositions are less varied and most seek to separate out a spin component. This can be achieved with emphasis placed on the electric field (as we do in this article), the magnetic field or on their average [5, 6]:

$$\mathbf{p} = \frac{1}{2c^2}\Re(\mathbf{E}^* \times \mathbf{H}) = \frac{\epsilon}{2\omega}\left[\Im(\mathbf{E}^* \cdot (\nabla)\mathbf{E}) + \frac{1}{2}\nabla \times \Im(\mathbf{E}^* \times \mathbf{E})\right] \equiv \mathbf{p}^{\mathbf{O}}_{\mathbf{E}} + \mathbf{p}^{\mathbf{S}}_{\mathbf{E}} \tag{1a}$$

$$\mathbf{p} = \frac{1}{2c^2}\Re(\mathbf{E}^* \times \mathbf{H}) = \frac{\mu}{2\omega}\left[\Im(\mathbf{H}^* \cdot (\nabla)\mathbf{H}) + \frac{1}{2}\nabla \times \Im(\mathbf{H}^* \times \mathbf{H})\right] \equiv \mathbf{p}^{\mathbf{O}}_{\mathbf{H}} + \mathbf{p}^{\mathbf{S}}_{\mathbf{H}} \tag{1b}$$

$$\mathbf{p} = \frac{1}{2}\left(\mathbf{p}^{\mathbf{O}}_{\mathbf{E}} + \mathbf{p}^{\mathbf{O}}_{\mathbf{H}}\right) + \frac{1}{2}\left(\mathbf{p}^{\mathbf{S}}_{\mathbf{E}} + \mathbf{p}^{\mathbf{S}}_{\mathbf{H}}\right) \equiv \mathbf{p}^{\mathbf{O}}_{\mathbf{E-H}} + \mathbf{p}^{\mathbf{S}}_{\mathbf{E-H}} \tag{1c}$$

The choice of gauge provides a further degree of freedom [7]. We interpret our results in terms of Eq. (1a), for the following reasons:

1. For the quasi-paraxial light beams we use here, orbital components of the momentum (e.g. $\mathbf{p}^{\mathbf{O}}_{\mathbf{E}}, \mathbf{p}^{\mathbf{O}}_{\mathbf{H}}, \mathbf{p}^{\mathbf{O}}_{\mathbf{E-H}}$) are very small or negligible in transverse directions. Thus, the spin components ($\mathbf{p}^{\mathbf{S}}_{\mathbf{E}}, \mathbf{p}^{\mathbf{S}}_{\mathbf{H}}, \mathbf{p}^{\mathbf{S}}_{\mathbf{E-H}}$) approximately equivalent in the transverse plane.

2. The force on a small, finite particle can be decomposed into parts that can be directly

identified with the underlying components of momentum (see Eqns. (9, 10)). This provides an immediate association with the decomposition Eq. (1a), and also with the Coulomb gauge which gives the force due to the orbital component of the momentum, $\mathbf{p}^{\mathbf{O}}_{\mathbf{E}}$ [7].

3. For Mie particles this formal association between spin force and spin momentum is lost. Nevertheless, a transverse force on a sphere requires a source of transverse momentum.

4. For dielectrics, the decomposition Eq. (1a) has been shown to make sense in a variety of experimental systems, including for spheres and slabs in the Mie regime [7–9].

5. $\mathbf{p}^{\mathbf{S}}_{\mathbf{E}}$ and $\mathbf{p}^{\mathbf{O}}_{\mathbf{E}}$ have been shown to couple to dielectrics in the Mie regime, in qualitatively different ways. For instance, consideration of the optical forces on finite flat plates indicate that $\mathbf{p}^{\mathbf{S}}_{\mathbf{E}}$ couples to matter via an edge effect [8].

Taken together, these points suggest that the decomposition Eq. (1a) is appropriate and physically meaningful not only for small particles but also for dielectric objects in the Mie regime. Additionally there is negligible quantitative difference between Eqns (1a-c) for near paraxial beams and the transverse forces we observe require a source of momentum which can reasonably be attributed to $\mathbf{p}^{\mathbf{S}}_{\mathbf{E}}$. For these reasons, we neglect the $\mathbf{E}$ and $\mathbf{H}$ subscripts throughout the main text and the rest of this document and refer generically to the spin, $\mathbf{p}^{\mathbf{S}} \equiv \mathbf{p}^{\mathbf{S}}_{\mathbf{E}}$ and orbital, $\mathbf{p}^{\mathbf{O}} \equiv \mathbf{p}^{\mathbf{O}}_{\mathbf{E}}$, components of the momentum.

B. Transverse Optical Momentum In Counter-Propagating Circularly Polarized Gaussian Beams

Decomposing the Poynting momentum via the electric field [5, 10], we have, in SI units:

$$\mathbf{p} = \frac{1}{2c^2}\Re\left(\mathbf{E}^* \times \mathbf{H}\right) = \frac{\epsilon_0}{2\omega}\Im\left[\mathbf{E}^* \cdot (\nabla)\mathbf{E}\right] + \frac{\epsilon_0}{4\omega}\nabla \times \Im\left[\mathbf{E}^* \times \mathbf{E}\right] \equiv \mathbf{p}^{\mathbf{O}} + \mathbf{p}^{\mathbf{S}}, \tag{2}$$

where c is the speed of light, and ϵ_0 the permittivity, in vacuum. The suffices $\mathbf{E}$ and $\mathbf{H}$ are suppressed from here on. The first part, $\mathbf{p}^{\mathbf{O}}$ is independent of polarization and referred to as orbital momentum. It is equivalent to the canonical momentum derived from Noethers theorem, expressed in the Coulomb gauge. The second term, $\mathbf{p}^{\mathbf{S}}$, is connected with inhomogeneous circular polarization and referred to as spin momentum. The ways in which

theses two contributions couple to matter is controversial. This is especially for the spin term whose appearance in Eq. (2) is a consequence of the symmetrization of the canonical stress-energy tensor [7, 11], and was previously thought virtual. Optical momentum in counter-propagating beams consists of the direct sum of the momenta associated with each independent beam, and an additional term associated with interference. Owing to the obvious symmetries in the geometry, the axial momentum of one beam effectively cancels that of the other. The remaining momentum consists of azimuthal components of spin and orbital momentum that circulate around the beam symmetry axis. Below we evaluate these components of the Poynting vector for single and counter-propagating beams, showing that the azimuthal momentum is dominated by the spin term, such that $p^{\mathrm{S}}_{\phi}/p^{\mathrm{O}}_{\phi} <\approx 2kz_{\mathrm{R}}$, where z_{R} is the Rayleigh range, related to the beam waist, W_0 by $z_{\mathrm{R}} = kw_0^2/2$. For the beams used in our experiment, $kz_{\mathrm{R}} \approx 150$.

Equation (3) gives the electric field in a circularly polarized beam, up to the first non-paraxial term.

$$\mathbf{E}^0 = \left[\hat{\mathbf{x}} + i\hat{\mathbf{y}} - \Big(\frac{x+iy}{q(z)}\Big)\hat{\mathbf{z}}\right]\frac{\mathbf{A}(\rho,z)}{\sqrt{2}}\exp\left(ikz\right) \tag{3}$$

With,

$$A(\rho,z) = A_0\frac{z_{\mathrm{R}}}{q(z)}\exp\Big(\frac{ik\rho^2}{2q(z)}\Big)$$

where $q(z) = z - iz_{\mathrm{R}}$.

In the Coulomb gauge the total, spin and canonical momentum are:

$$\mathbf{p} = \frac{\epsilon_0}{2\omega}\Im\big(\mathbf{E}^* \times \nabla \times \mathbf{E}\big) \tag{4a}$$

$$\mathbf{p}^{\mathbf{O}} = \frac{\epsilon_0}{2\omega}\Im\big(\mathbf{E}^* \cdot (\nabla)\mathbf{E}\big) \tag{4b}$$

$$\mathbf{p}^{\mathbf{S}} = \frac{\epsilon_0}{4\omega}\nabla \times \Im\big(\mathbf{E}^* \times \mathbf{E}\big) \tag{4c}$$

For a single beam, the azimuthal components of the spin and canonical momenta are:

$$p_\phi^{0,\mathrm{O}} = \frac{\epsilon_0}{2\omega}\frac{|E_z^0|^2}{\rho}, \tag{5a}$$

$$p_\phi^{0,\mathrm{S}} = \frac{\epsilon_0}{2\omega}\left[\frac{2k\rho z_\mathrm{R}|E_{x/y}^0|^2}{z^2+z_\mathrm{R}^2} + \frac{2\Re(f)\rho z|E_{x/y}^0|^2}{z^2+z_\mathrm{R}^2} - \frac{(z^2-z_\mathrm{R}^2)\rho|E_{x/y}^0|^2}{(z^2+z_\mathrm{R}^2)^2}\right], \tag{5b}$$

$$\approx \frac{\epsilon_0}{2\omega}\left[\frac{2k\rho z_\mathrm{R}|E_{x/y}^0|^2}{z^2+z_\mathrm{R}^2}\right] \tag{5c}$$

With $f = f(\rho, z) = ik - \frac{1}{q(z)}\left(1 + \frac{ik\rho^2}{2q(z)}\right)$. The second two factors on the right of Eq. (5b) are negligible (smaller by a factor of $\approx kz_R$). Combining Eq. (5) with Eq. (3) gives,

$$\frac{p_\phi^{0,\mathrm{S}}}{p_\phi^{0,\mathrm{O}}} = 2kz_\mathrm{R} \tag{6}$$

Thus, as the beams are defocused, and the Rayleigh range increased, the ratio of the spin to canonical momentum in a single circularly polarized Gaussian beam increases in proportion to kz_R; this ratio is independent of position within the beams.

Counter-propagating beams contain a series of interference fringes which modulate the intensity and momentum fields of isolated beams. For a counter-propagating system with an electric field described by the sum of Eq. (3) with its reflection through the $z = 0$ plane, the modulus of the electric field is:

$$|\mathbf{E}^\mathrm{tot}|^2 \approx 4|\mathbf{E}^0|^2\cos^2(kz), \tag{7}$$

so that bright fringes appear at $z \approx 0, \lambda/2, \lambda, 3\lambda/4....$ Eq. (7) is a good approximation for near-paraxial beams. However, it fails qualitatively in one important respect. Counter-propagating plane waves contain dark fringes for which the intensity vanishes on extended planes. Even weak focusing destroys this condition. In the dark fringes of near-paraxial counter-propagating waves, the intensity approaches zero, but reaches this only at discrete points on the beam axis. Including the appropriate interference terms the azimuthal spin and canonical momentum are, to lowest order:

$$p_\phi^{\mathrm{tot,S}} \approx 2p_\phi^{0,\mathrm{S}}, \tag{8a}$$

$$p_\phi^{\mathrm{tot,O}} \approx 4p_\phi^{0,\mathrm{O}}\sin^2(kz), \tag{8b}$$

$$\frac{p_\phi^{\mathrm{tot,S}}}{p_\phi^{tot,\mathrm{O}}} \approx \frac{kz_\mathrm{R}}{\sin^2(kz)}. \tag{8c}$$

Thus, in near-paraxial, counter-propagating, circularly polarized Gaussian beams, the mean ratio of transverse spin to transverse canonical momentum is $2kz_{\mathrm{R}}$, with the canonical component becoming small in the bright interference fringes.

Although these equations provide a good approximation to the momenta, they suggest an intriguing paradox. The canonical momentum falls to zero in each bright interference fringe, but reaches a maximum in each dark fringe. This apparent irregularity is resolved by observing that the wave-front curvature prevents the intensity falling to zero in a dark fringe except at discrete points on the axis, where the azimuthal momentum also vanishes. Nevertheless, the canonical momentum density in the dark fringes can become surprisingly large, even though its absolute value remains small. Additionally, it should be noted that, whilst the azimuthal canonical momentum is susceptible to strong interference effects, the azimuthal spin of the counter-propagating beams is approximately the sum of that for the two separate beams with interference having a marginal effect.

Throughout the beam, the azimuthal spin momentum is larger than the canonical momentum by a factor of at least kz_{R} (≈ 150 for our beams), the mean ratio being ≈ 300. Numerical evaluation of the Richards-Wolf representation of the counter-propagating beams shows that the ratio of the momenta in a bright fringe is $\approx (kz_{\mathrm{R}})^2$, Fig. (1).

Supplementary Note 2. OPTICAL FORCES

A. Small particle approximation

The spin and orbit (or, canonical) components of the Poynting momentum couple to light in fundamentally different ways. For instance, the optical pressure applied to a flat plate by $\mathbf{p^S}$ does not scale with the exposed area of the plate, suggesting that coupling of $\mathbf{p^S}$ is an edge effect [8]. Similarly, the force on a small particle manifests as a finite size effect [7]. To lowest order, the optical scattering force on a polarizable point particle is directly proportional to $\mathbf{p^O}$ [7]:

$$\mathbf{F}^0 = c^3 \Im\left[\alpha_{\mathrm{e}}\right] \mathbf{p^O}. \tag{9}$$

Optical spin forces appear at the next highest order [7, 12]:

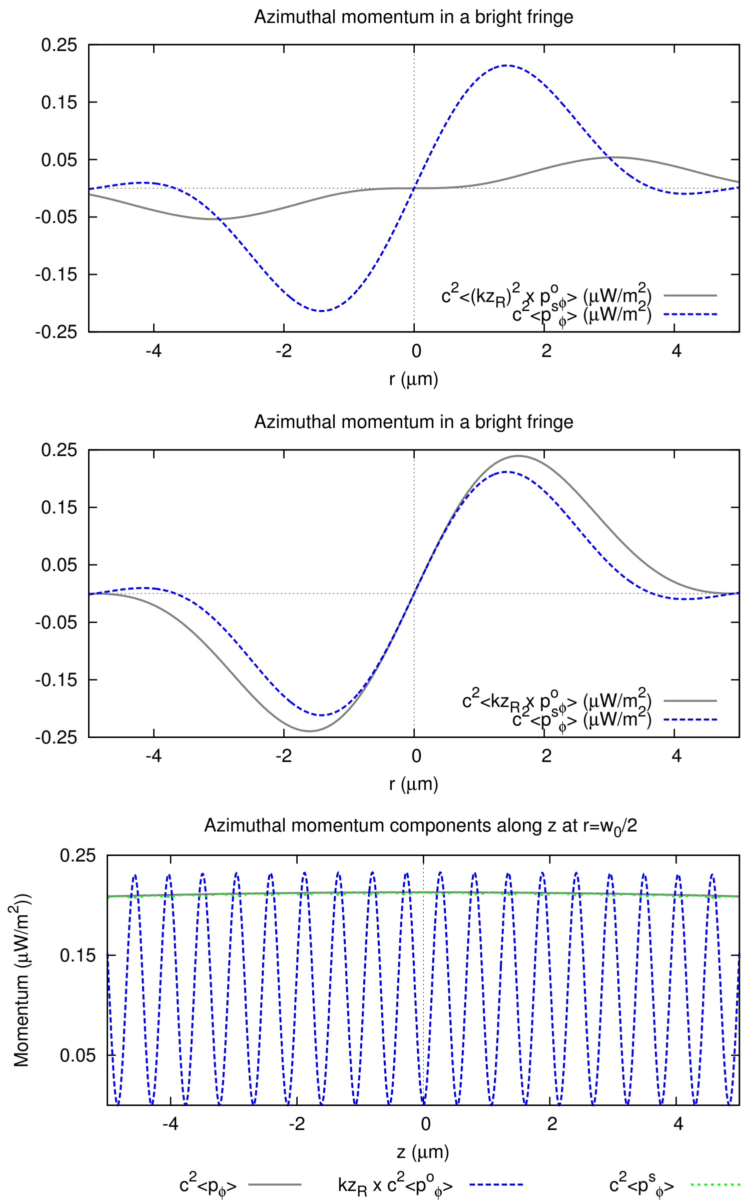

Supplementary Figure 1. Variation of the azimuthal momentum with the radial coordinate in counter-propagating circularly polarized Gaussian beams, in a bright fringe (a), and a dark fringe (b). The variation of azimuthal momenta with axial coordinate at a radius, $\rho = W_0/2$ is given in (c). Calculations performed under the Richards-Wolf representation

$$\mathbf{F}^1 = -\frac{\omega^3}{3}\Re\left[\alpha_{\mathrm{e}}\alpha_{\mathrm{m}}^*\right]\left(\mathbf{p}^{\mathbf{O}} + \mathbf{p}^{\mathbf{S}}\right). \tag{10}$$

$\alpha_{\mathrm{e/m}}$ is the electric/magnetic polarizability which, for dielectric particles are, approximately:

$$\alpha_{\mathrm{e/m}} = \alpha^0_{\mathrm{e/m}}/(1 - \frac{2}{3} i k^3 \alpha^0_{\mathrm{e/m}}) \tag{11a}$$

$$\alpha^0_{\mathrm{e}} \approx \frac{\epsilon_{\mathrm{m}}}{k^3}\left[\frac{(\epsilon_{\mathrm{p}} - \epsilon_{\mathrm{m}})(ka)^3}{(\epsilon_{\mathrm{p}} + 2\epsilon_{\mathrm{m}})}\right] \tag{11b}$$

$$\alpha^0_{\mathrm{m}} \approx \frac{(\epsilon_{\mathrm{p}} - \epsilon_{\mathrm{m}})(ka)^5}{30 k^3 \epsilon_{\mathrm{m}}} \tag{11c}$$

Here, $\alpha^0_{\mathrm{e/m}}$ are the Claussius-Mossotti polarizabilities for dielectric particles with permeability equal to one. $\alpha_{\mathrm{e/m}}$ are the polarizabilties, corrected for finite size. These latter polarizabilities contain an imaginary part, even for non-absorbing particles, that produces the scattering force captured by Eq. (9).

Comparing Eq. (9) and Eq. (10), the ratio of the force due to $\mathbf{p^S}$ to that due to $\mathbf{p^S}$ is:

$$\frac{\mathbf{F}^1}{\mathbf{F}^0} \approx -\frac{k^3}{3}\frac{\Re[\alpha_{\mathrm{e}}\alpha^*_{\mathrm{m}}]}{\Im[\alpha_{\mathrm{e}}]}\frac{\mathbf{p^S}}{\mathbf{p^O}} = -(ka)^2 \frac{(\epsilon_{\mathrm{p}} + 2\epsilon_{\mathrm{m}})}{30\epsilon^2_{\mathrm{m}}}\frac{\mathbf{p^S}}{\mathbf{p^O}} \approx -\frac{(ka)^2}{14}\frac{\mathbf{p^S}}{\mathbf{p^O}}, \tag{12}$$

where it is assumed $\mathbf{p^S} \gg \mathbf{p^O}$, as discussed above. The term on the far left corresponds to the case of a silica particle ($\epsilon_{\mathrm{p}} = 2.1$) in vacuum. Thus, as the particle size increases, spin forces begin to dominate. In accordance with Eq. (10), these forces act in the opposite direction to the Poynting vector. Figure (2) shows this transition. For a very small particle (Fig. 2, top panel), the transverse force is almost directly proportional to $\mathbf{p^O}$, and has the same variation with the axial coordinate, z. In this regime, the force is in the same direction as the Poynting vector. As the particle size increases, coupling with $\mathbf{p^S}$ strengthens until the force is a approximately independent of z, and acts in the direction opposing the Poynting momentum (Fig. 2, lower panels).

B. Mie particles

Optical forces are evaluated under the generalized Lorentz-Mie theory, using the Maxwell Stress Tensor (MST). The MST quantifies the momentum flux, assuming the classical Poynting momentum. The beam is modelled using the Richards-Wolf description of a Gaussian beam. The beam is reflected through a plane perpendicular to the axis, passing through the focal point, to give the counter-propagating beam used in the experiments. Figure (3) shows radial and azimuthal forces and their variantions both along the beam axis (in the z direction) and in the radial direction. It should be noted that both force components vary only

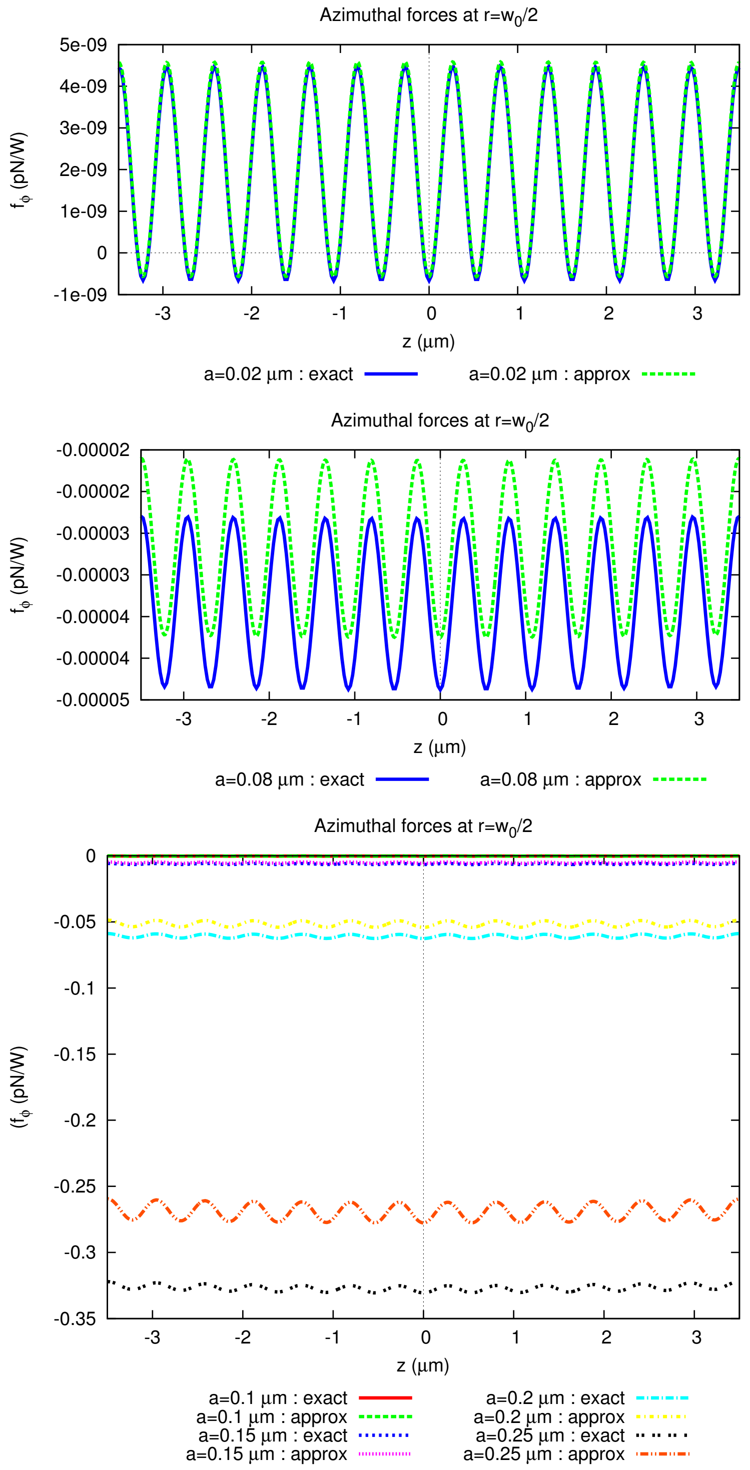

Supplementary Figure 2. Azimuthal forces on small particles of increasing size. Approximate results using Eqns. 9 and 10 are plotted with exact results using the Mie theory described below.

weakly with z, and that the ratio $f_\phi^{\rm opt}/f_r^{\rm opt} \approx 0.17$ is approximately constant throughout the trap.

Supplementary Note 3. LANGEVIN EQUATION OF MOTION

In general, the motion of the spherical bead is given by a Langevin equation (see, for example, [13]), Eq. (13).

$$\mathbf{F}^{\rm opt}(\mathbf{r}) - \xi\dot{\mathbf{r}} + \mathbf{F}^{\rm L}(t) = m\ddot{\mathbf{r}} \tag{13}$$

$\mathbf{F}^{\rm opt}$ is the absolute optical force as a function of position, $\mathbf{r}$. For a particular beam, all of the optical forces are directly proportional to the total optical power. Elsewhere, lower case letters will be used to indicate the power per unit power e.g. $\mathbf{F}^{\rm opt} = P\mathbf{f}^{\rm opt}$. $\mathbf{F}^{\rm L}(t)$ is an uncorrelated fluctuation, Eq. (14).

$$\langle \mathbf{F}^{\rm L}(t)\rangle = 0 \tag{14a}$$

$$\langle \mathbf{F}^{\rm L}(t) \otimes \mathbf{F}^{\rm L}(t')\rangle = 2k_{\rm B}T\xi\mathbf{I}\delta(t-t') \tag{14b}$$

Where $\xi = 6\pi\mu a$ is the usual Stokes coefficient for a sphere of radius a and $\mathbf{I}$ is a unit matrix.

Under vacuum conditions the viscosity, μ is a function of the ambient pressure [14], Eq. (15).

$$\mu = \mu_0 \left(\frac{0.619}{0.619 + K_{\rm n}}\right)(1 + c_{\rm K}) \tag{15}$$

$\mu_0 = 1.9 \times 10^{-5}$ Pa s is the viscosity of air at atmospheric pressure, $K_{\rm n} = \bar{l}/a$ the Knudsen number and $c_{\rm K}$ a factor:

$$c_{\rm K} = \frac{0.131K_{\rm n}}{0.785 + 1.152K_{\rm n} + K_{\rm n}^2} \tag{16}$$

The pressure dependence enters via the mean free path as follows:

$$\bar{l} = \frac{p_0}{p_{\rm a}}\bar{l}_0 \equiv p_{\rm r}\bar{l}_0 \tag{17}$$

where p_0 is atmospheric pressure, and $p_{\rm a}$ is the actual pressure in the chamber.

In what follows, we prefer to use measured values of the drag coefficients, see section Supplementary Note 5.

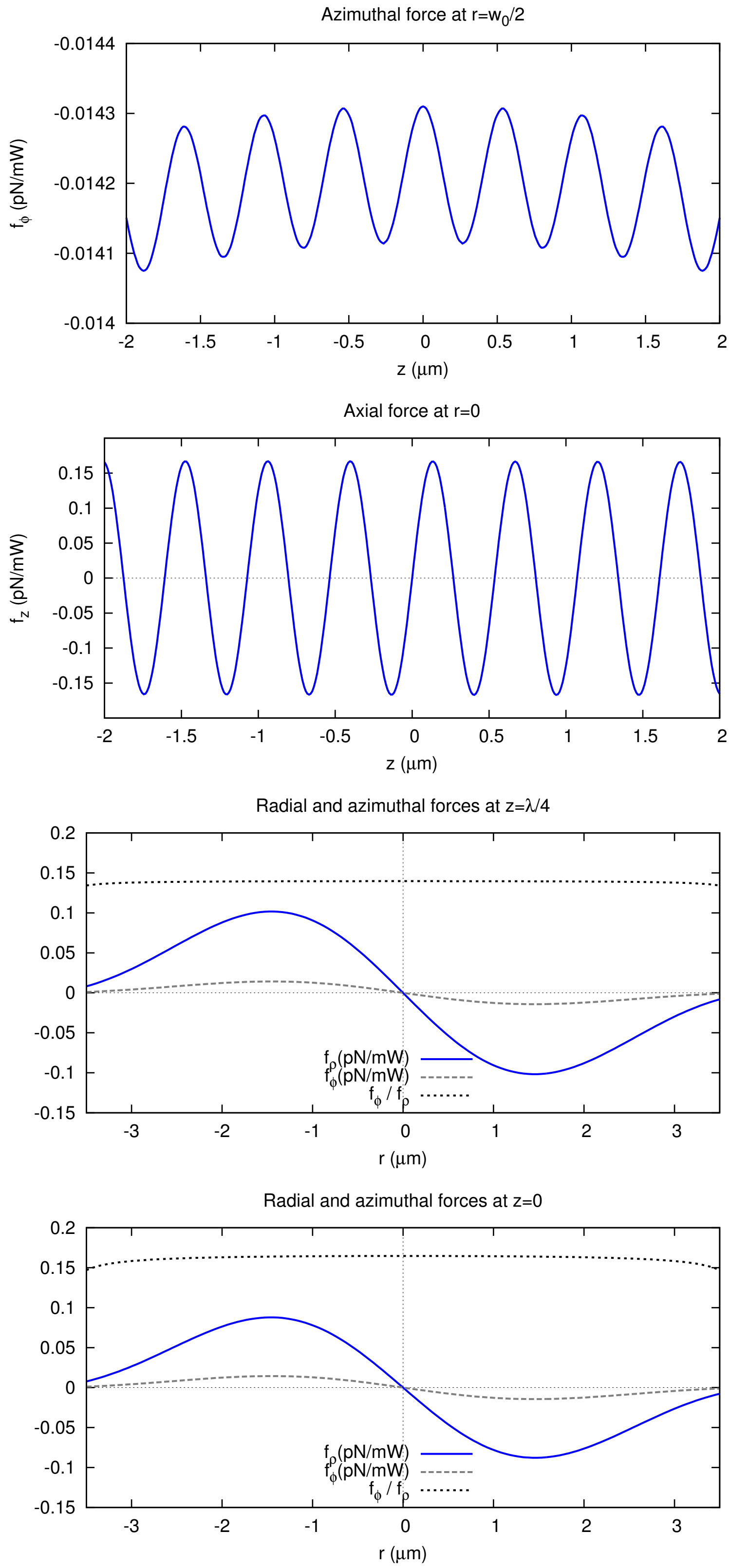

Supplementary Figure 3. (a) Variation of the azimuthal force with the axial coordinate, at a radial distance $r = W_0/2$, radial and azimuthal forces at $z = \lambda/4$ (b), and $z = 0$ (c)

A. Linear Regime, Characteristic Frequencies

For small displacements, the optical force field can be linearized about the mechanical equilibrium position, on the beam axis. In Cartesian coordinates,

$$\mathbf{F}^{\text{opt}} = -\mathbf{K}\mathbf{r},$$
$$\mathbf{K} = \begin{bmatrix} K_r & K_\phi \\ -K_\phi & K_r \end{bmatrix}. \tag{18}$$

Where, $\mathbf{K}$ is a non-symmetric two dimensional tensor given by the sum of a isotropic diagonal part, $K_r\mathbf{I}$, associated with the conservative gradient force, and a skew symmetric part, with entry K_ϕ, associated with the non-conservative spin force. K_ϕ can be positive or negative, depending on the handedness of the circular polarization. The power normalised stiffness tensor is written, $\mathbf{k}$ with spin and gradient coefficients, k_ϕ, k_r so that $\mathbf{F}^{\text{opt}} = P\mathbf{f}^{\text{opt}} = -\mathbf{K}\mathbf{r} = -P\mathbf{k}\mathbf{r}$. The linearized equation of motion is then:

$$-\mathbf{K}\mathbf{r} - \xi\dot{\mathbf{r}} + \mathbf{F}^{\text{L}} = m\ddot{r} \tag{19}$$

Or, in the frequency domain:

$$\Big(-\mathbf{K} - i\omega\xi + m\omega^2\Big)\mathbf{R} = -\widetilde{\mathbf{F}}^{\text{L}} \tag{20a}$$

$$\langle\widetilde{\mathbf{F}}^{\text{L}}(\omega) \otimes \widetilde{\mathbf{F}}^{\text{L},*}(\omega')\rangle = 2k_{\text{B}}T\xi\delta(\omega - \omega'). \tag{20b}$$

where $\mathbf{R}$ is the Fourier transform of the Cartesian coordinates of position, $\mathbf{r}$.

The motion of the trapped particle can be described in terms of characteristic frequencies, $\omega = \omega_c$, obtained by setting the determinant of the left hand side matrix, Eq. (20a) to zero e.g.

$$|-\mathbf{K} - i\xi\omega_c + m\omega_c^2| = 0 \Rightarrow \tag{21a}$$

$$\omega_c = \pm\frac{1}{2m}\sqrt{-\xi^2 + 4m(K_r \pm iK_\phi)} + i\frac{\xi}{2m} \tag{21b}$$

$$\approx \pm\omega_0 + \frac{i}{2m}\left(\xi \pm \frac{K_\phi}{\omega_0}\right). \tag{21c}$$

Where $\omega_0 = \sqrt{K_r/m}$ is the conventional resonant frequency of a harmonic oscillator with spring constant, K_r. There are four distinct values of ω_c, corresponding to the different permutations of the $\pm$ signs. To simplify the notation we write,

$$\begin{aligned}\Pi &= \sqrt{-\xi^2 + 4m(K_r + iK_\phi)} \\ &\approx 2m\omega_0 + i\frac{K_\phi}{\omega_0},\end{aligned} \tag{22}$$

and,

$$\omega_{c+} = (\Pi + i\xi)/2m \tag{23a}$$

$$\omega_{c-} = (-\Pi + i\xi)/2m. \tag{23b}$$

Then, the full set of characteristic frequencies are:

$$\{\omega_c\} = \{\omega_{c+}, -\omega_{c+}^*, \omega_{c-}, -\omega_{c-}^*\} \tag{24}$$

In general, the real parts of the ω_c relate to oscillatory motion, and the imaginary parts either to growth or decay, depending on the sign. The exact expression for ω_c, Eq. (21b) reveals a fundamental difference between motion in a linearly polarized trap, where $K_\phi = 0$, and a circularly polarized trap where $K_\phi \neq 0$. We discuss these cases separately below. **Linear polarization, $K_\phi = 0$:** In this case, the ω_c are degenerate with $\omega_{c+} = -\omega_{c-}^*$ and $\omega_{c-} = -\omega_{c+}^*$, leaving two unique values, ω_{c+} and ω_{c-}. In the limit of low optical power (or high viscosity), $\xi^2 \gg 4mK_r$ so the Π and, therefore, ω_c is purely imaginary and the motion consists of relaxation only. This is the over-damped regime. As the optical power is increased, $K_r = Pk_r$ increases and the first term becomes positive when $4mK_r > \xi^2$ and ω_c acquires a real component, corresponding to oscillatory behaviour. In this, under-damped regime, the motion corresponds to a damped oscillation at frequency a $\omega \approx \sqrt{K_r/m}$ that increases with $\sqrt(P)$, with an invariant decay constant, $\xi/2m$.

Circular polarization, $K_\phi \neq 0$: In this case Π, Eq. (22), is generally complex and there are four distinct ω_c. Since $\Pi \propto \sqrt{P}$, the ω_c are similar to those for the $K_\phi = 0$ case when P is small e.g. for small P, $\omega_c \approx \pm\sqrt{K_r/m} + i\xi/2m$. As the optical power is increased both the real and imaginary parts deviate increasingly from these values. Most significantly, the imaginary parts of two of the ω_c approach zero with increasing optical power. In particular, for $K_\phi > 0$, $\Im(\omega_{c-})$ approaches zero, and is equal to it when the optical power, P reaches a threshold value $P = P_{\text{thr}}$.

$$\frac{P_{\text{thr}}}{\xi^2} = \frac{k_r}{mk_\phi^2} \tag{25a}$$

$$\frac{\Omega_{\text{thr}}}{\xi} = \frac{k_r}{mk_\phi} \tag{25b}$$

Where Ω_{thr} is the value of $\Re(\omega_{\text{c}-})$, the oscillatory response of the system, at $P = P_{\text{thr}}$. Equivalently, $\Im(\omega_{\text{c}+})$ approaches zero and $\Re(\omega_{\text{c}+}) \to \Omega_{\text{thr}}$ at P_{thr} when $K_\phi < 0$. Since $\Im(\omega_{\text{c}})$ quantifies the time constant for the damped oscillations, this process signifies a transition to sustained, driven vibration in which the viscous drag of the ambient gas is compensated for by the driving force, in this case provided by optical spin momentum.
The thermal motion of the particle, particularly the distribution of the particle energy in frequency space, can be understood through the generalised power spectrum, described below.

B. Power Spectrum

In terms of the optical force field, the beam axis is a stable equilibrium position, where the forces vanish and are locally restoring. In this configuration, the particle is exposed to thermal fluctuations that take the form of white noise. The amplitude of the resulting motion depends on the susceptibility of the trap, which is frequency dependent. Obviously, thermal forces operating at frequencies at, or close to, the characteristic frequencies described above, will have a greater effect on the motion. When the optical power is such a characteristic frequency is purely real or has a small imaginary part, high amplitude motion and instability will result. These factors are quantified through the power spectrum.

From Eq.(20), we have

$$\mathbf{R} = -\left(-\mathbf{K} - i\xi\omega + m\omega^2\right)^{-1}\mathbf{F}^{\text{L}} \equiv -\mathbf{M}\mathbf{F}^{\text{L}}. \tag{26}$$

Combining with Eq. (14), gives :

$$\langle \mathbf{R}(\omega)\mathbf{R}^\star(\omega)\rangle = 2k_{\text{B}}T\xi\mathbf{M}(\omega)\mathbf{M}^H(\omega) \tag{27}$$

Where the superscript, H, indicates the Hermitian transpose and,

$$\mathbf{M} = \frac{1}{(-K_r + m\omega^2 - i\xi\omega)^2 + K_\phi^2}\begin{bmatrix} (-K_r + m\omega^2 - i\xi\omega) & K_\phi \\ -K_\phi & (-K_r + m\omega^2 - i\xi\omega) \end{bmatrix} \tag{28}$$

The power spectral density is:

$$\langle \mathbf{R}(\omega)\mathbf{R}^\star(\omega)\rangle = \frac{2k_{\mathrm{B}}T\xi}{\left[(K_r-\omega^2 m)^2+\omega^2\xi^2\right]^2+2K_\phi^2\left[(K_r-\omega^2 m)^2-\omega^2\xi^2\right]+K_\phi^4}\times \begin{bmatrix} (K_r-\omega^2 m)^2+\omega^2\xi^2+K_\phi^2 & 2i\xi\omega K_\phi \\ -2i\xi\omega K_\phi & (K_r-\omega^2 m)^2+\omega^2\xi^2+K_\phi^2 \end{bmatrix}. \tag{29}$$

This can be expanded in the characteristic frequencies, ω_{c}. The factor $\mathbf{M}^{\mathrm{H}}$ is associated with another set of characteristic frequencies, given simply by the negative values, $-\omega_{\mathrm{c}}$, leading to,

$$\langle \mathbf{R}(\omega)\mathbf{R}^\star(\omega)\rangle = \frac{2k_{\mathrm{B}}T\xi}{m^2\prod_i(\omega^2-\omega_{c,i}^2)}\begin{bmatrix} (\omega^2-\nu_+^2)(\omega^2-\nu_-^2)+K_\phi^2/m^2 & -2i\xi\omega K_\phi/m^2 \\ 2i\xi\omega K_\phi/m^2 & (\omega^2-\nu_+^2)(\omega^2-\nu_-^2)+K_\phi^2/m^2 \end{bmatrix}. \tag{30}$$

Where $\omega_{\mathrm{c},i}$ label the characteristic frequencies, ω_{c} and $\nu_\pm$ are the characteristic frequencies when $K_\phi = 0$, i.e.,

$$\begin{aligned}\nu_\pm &= \pm\frac{1}{2m}\sqrt{-\xi^2+4mK_r}+i\frac{\xi}{2m}\\ &\approx \pm\omega_0+i\frac{\xi}{2m}\end{aligned} \tag{31}$$

For brevity we write,

$$\nu_\pm = (\pm\Lambda + i\xi)/2m \tag{32}$$

with $\Lambda = \sqrt{-\xi^2+4mK_r}$. As with the previous discussion of the characteristic frequencies, the power spectrum depends qualitatively on whether or not $K_\phi = 0$. We discuss these two cases separately, below.

Linear polarization, $K_\phi = 0$: In this case, the cross terms in $\langle \mathbf{R}(\omega)\mathbf{R}^*(\omega)\rangle$ disappear, and the characteristic frequencies are degenerate, leaving only two independent values, $\omega_{\mathrm{c}+}$ and $\omega_{\mathrm{c}-}$, equivalent to $\nu_\pm$ when $K_\phi = 0$. The power spectrum consists only of the diagonal terms $\langle X(\omega)X^*(\omega)\rangle = \langle Y(\omega)Y^*(\omega)\rangle$, which takes the familiar form of a Lorentzian:

$$\langle X(\omega)X^*(\omega)\rangle = \frac{2k_{\mathrm{B}}T\xi}{m^2\big(\omega^2-\nu_+^2\big)\big(\omega^2-\nu_-^2\big)}. \tag{33}$$

This function consists of a single peak, which attains its maximum when $\omega^2 = \Re(\nu_+^2) = \Re(\nu_-^2)$. In the under-damped regime, this is $\omega \approx \sqrt{K_r/m}$, and the peak height is,

$$\langle X(\omega)X^*(\omega)\rangle_{\max} = \frac{2k_{\rm B}T}{\xi\omega_0^2} \propto \frac{1}{P}. \tag{34}$$

As usual, the peak width at half the maximum is given by the imaginary part of $\omega_{\rm c}$, i.e. $\xi/2m$.

Circular polarization, $K_\phi \neq 0$ **:** In this case, the cross terms in the power spectrum, $\langle X(\omega)Y^*(\omega)\rangle = \langle Y(\omega)X^*(\omega)\rangle$ do not vanish, and all four characteristic frequencies are distinct. As described above, we expect the imaginary parts of two of the characteristic frequencies to approach zero as the optical power is increased towards $P_{\rm thr}$: for $K_\phi > 0$, $\Im(\omega_{\rm c-}) \to 0$ (and $-\omega_{\rm c-}^* \to 0$) as $P \to P_{\rm thr}$. When $K_\phi < 0$, $\omega_{\rm c+} \to 0$. The power dependence of the power spectrum can be appreciated through the approximate form of the characteristic frequencies, Eq. (21c) which, in terms of the optical power, P, can be written:

$$\omega_{\rm c} \approx \pm\omega_0 + \frac{i\xi}{2m}\left(1 \pm \sqrt{\frac{P}{P_{\rm thr}}}\right), \tag{35}$$

where $P_{\rm thr}$ is the threshold power, Eq. (25a). For low optical power, ω_c are close to the characteristic frequencies when $K_\phi = 0$ (i.e. $\omega_{\rm c\pm} \approx \nu_\pm$) and the dependence of the power spectrum on the optical power is similar to that in the case of linear polarization i.e. the peak height decreases according to $\approx 1/P$, and the width remains approximately constant. As P increases towards $P_{\rm thr}$, this behaviour changes qualitatively. Writing $\delta = (P - P_{\rm thr})$, the characteristic frequencies are approximately,

$$\omega_{\rm c} \approx \pm\omega_0 + \frac{i\xi}{2m}\Big(1 \pm \big(1 + \frac{\delta}{2P_{\rm thr}}\big)\Big), \tag{36}$$

and the peak heights of the diagonal terms in the power spectrum are approximately,

$$\langle X(\omega)X^*(\omega)\rangle_{\max} \approx \frac{2k_{\rm B}T(\xi^2\omega_0^2 + K_\phi^2)P_{\rm thr}^2}{\xi^3\omega_0^4\delta^2} \approx \frac{2k_{\rm B}TP_{\rm thr}^2}{\xi\omega_0^2\delta^2} \propto \frac{1}{\delta^2}. \tag{37}$$

As the optical power, P, approaches the threshold power, $P_{\rm thr}$ the peak in the power spectrum grows in height according to $1/(P - P_{\rm thr})^2$ and decreases in width according to the imaginary part of $\Im(\omega_{\rm c}) \propto (P - P_{\rm thr})$.

In summary, for linearly polarized beams, the peak height in the power spectrum decreases with optical power, $\propto 1/P$ and the width remains constant. For circularly polarized beams,

the same behaviour is obtained when $P \ll P_{\text{thr}}$ but, as P approaches P_{thr} the peak height grows as $1/(P - P_{\text{thr}})^2$ while the width decreases with $(P - P_{\text{thr}})$.

C. Variance And Correlations

According to the Wiener-Khinchine theorem, time correlations and particle variance follow from the Fourier transform of the power spectrum [13].

$$\langle \mathbf{r}(t)\mathbf{r}(t+\tau)\rangle = \frac{1}{2\pi}\int_{-\infty}^{\infty}\langle \mathbf{R}(\omega)\mathbf{R}^{\star}(\omega)\rangle e^{-i\omega\tau}d\omega \tag{38}$$

For $\tau = 0$ these quantities correspond to the variance of the particle in the trap, and for $\tau > 0$ we have the correlation between the position of the particle between present and future times. Eq. (38) can be evaluated with the residue theorem, using a hemi-circular contour, closed in the lower half plane for $\tau > 0$,

$$\langle \mathbf{r}(t)\mathbf{r}(t+\tau)\rangle = \frac{1}{2\pi}\oint\langle \mathbf{R}(\omega)\mathbf{R}^{\star}(\omega)\rangle e^{-i\omega\tau}d\omega = i\sum_i \text{Res}(\langle \mathbf{R}(\omega)\mathbf{R}^{\star}(\omega)\rangle e^{-i\omega\tau}, \omega_{\text{c},i}) \tag{39}$$

Where the sum is taken over the poles lying in the lower half plane. Comparing the case of linear and circularly polarized beams leads to the following conclusions.

Linear polarization, $K_\phi = 0$ **:** Summing the residues gives, for the diagonal terms,

$$\begin{aligned}\langle x(t)x(t+\tau)\rangle &= \frac{k_{\text{B}}T}{\Lambda K_r}\big(\Lambda\cos(\Lambda\tau/2m) - \xi\sin(\Lambda\tau/2m)\big)e^{-\xi\tau/2m}\\ &\approx \frac{k_{\text{B}}T}{\omega_0 K_r}\Big(\omega_0\cos(\omega_0\tau) + \frac{\xi}{2m}\sin(\omega_0\tau)\Big)e^{-\xi\tau/2m}.\end{aligned} \tag{40}$$

When $\tau = 0$, $\langle x^2\rangle = k_{\text{B}}T/K_r$ which follows directly from equipartition, by equating the elastic energy of the trap, $\frac{1}{2}K_r\langle x^2\rangle$ with the thermal energy for a single degree of freedom, $\frac{1}{2}k_{\text{B}}T$. In the under-damped regime, the approximation of $\Lambda/2m \approx \omega_0$ is accurate. In addition, the second term, with the sinusoidal variation is negligibly small. The thermal motion of a particle in a linearly polarized trap therefore consists of oscillations at the resonant frequency, which decorrelate exponentially, with a time constant determined by the viscous drag and the mass. Increasing the optical power decreases the amplitude of the oscillations, increases the frequency, but leaves the coherence time unaltered.

Circular polarization, $K_\phi = 0$ **:** Properties of the covariance matrix, Eq. (39) can be appreciated by examining the residues of the four poles captured by the contour. For $\tau = 0$, they have the following form:

ω_c	Res($\langle XX\rangle$)	Res($\langle XY\rangle$)
ω_{c+}	$a+ib$	$i(a+ib) = \quad -b+ia$
ω_{c-}	$A-ib$	$i(A-ib) = \quad +b+iA$
$-\omega^*_{c+}$	$a-ib$	$-i(a-ib) = -b-ia$
$-\omega^*_{c-}$	$A+ib$	$-i(A+ib) = +b-iA$
Sum	2(a+A)	0

Where a, A and b are functions of optical power (through the absolute stiffness coefficients). This general form follows directly from the construction of the power spectral density matrix, $\langle \mathbf{R}(\omega)\mathbf{R}^*(\omega)\rangle$, Eq. (30), and the formulation of the characteristic frequencies. Several features should be noted. First, the residues for the diagonal and cross terms are related by, $\text{Res}(\langle XX\rangle) = \pm i\text{Res}(\langle XY\rangle)$. This causes the causes the sum of the residues for the cross term to vanish, ensuring that the x and y coordinates are instantaneously uncorrelated, as they should be e.g. $\langle xy\rangle = 0$. This also fixes the relative phases of the oscillatory parts of $\langle x(t+\tau)x(t)\rangle$ and $\langle x(t+\tau)y(t)\rangle$, as described below. Second, either $A(P)$ or $a(P)$ are singular for $P \to P_{\text{thr}}$, depending on the sign of K_ϕ. For example, when $K_\phi > 0$, $\Im(\omega_{c-}) \to 0$ as $P \to P_{\text{thr}}$. Under this condition, the residues for ω_{c-} and its complex conjugate contain a factor, $(\omega_{c-}^2 - (\omega_{c-}^*)^2)$. From the approximate form of ω_{c-}, Eq. (36) this evaluates to, $(\omega_{c-}^2 - (\omega_{c-}^*)^2) \approx -\frac{i\xi\omega_0\delta}{2mP_{\text{thr}}}$, where $\delta = (P - P_{\text{thr}})$, so that $A(P) \propto 1/\delta$ as P approaches P_{thr}. With these considerations in mind, the time covariance of the coordinates is:

$$\begin{aligned}\langle x(t+\tau)x(t)\rangle \approx \exp\left[-\big(\xi + K_\phi\sqrt{\frac{m}{K_r}}\big)\tau/2m\right]\big[a\cos(\omega_0\tau) - b\sin(\omega_0\tau)\big]+ \\ \exp\left[-\big(\xi - K_\phi\sqrt{\frac{m}{K_r}}\big)\tau/2m\right]\big[A\cos(\omega_0\tau) - b\sin(\omega_0\tau)\big],\end{aligned} \tag{41}$$

for the diagonal terms, and for the cross terms,

$$\begin{aligned}\langle x(t+\tau)y(t)\rangle \approx \exp\left[-\big(\xi + K_\phi\sqrt{\frac{m}{K_r}}\big)\tau/2m\right]\big[-a\cos(\omega_0\tau) + b\sin(\omega_0\tau)\big]+ \\ \exp\left[-\big(\xi - K_\phi\sqrt{\frac{m}{K_r}}\big)\tau/2m\right]\big[b\cos(\omega_0\tau) - A\sin(\omega_0\tau)\big],\end{aligned} \tag{42}$$

Where the approximate form of ω_c, valid in the under-damped regime, has been used

(Eq. (21). As the optical power is increased towards $P_{\rm thr}$, singular terms ($A(P)$ for $K_\phi > 0$) dominate and the corresponding decay constants increase rapidly leaving:

$$\langle x(t+\tau)x(t)\rangle \approx A\exp\left[\xi\delta\tau/4mP_{\rm thr}\right]\cos(\omega_0\tau) \tag{43a}$$

$$\langle x(t+\tau)y(t)\rangle \approx -A\exp\left[\xi\delta\tau/4mP_{\rm thr}\right]\sin(\omega_0\tau), \tag{43b}$$

where the approximation, Eq. (36), for $\delta = (P - P_{\rm thr}) < 0$ has been used. Thus, as the optical power approaches $P_{\rm thr}$, the covariance of the coordinates consist of a damped oscillation with amplitude $\propto 1/(P_{\rm thr} - P)$ at the resonant frequency, ω_0, decaying with a long time constant $\propto 1/(P_{\rm thr} - P)$. In other words, the particle motion undergoes a transition from biased stochastic motion towards deterministic, sustained oscillation.

The phase difference between the oscillations, $\langle x(t+\tau)y(t)\rangle$ and $\langle x(t+\tau)x(t)\rangle$ indicates sustained circular motion. When $P = P_{\rm thr}$, the frequency of this motion is given by $\Omega_{\rm thr}$, Eq. (25b). For yet higher optical powers, the particle begins to orbit the beam axis deterministically, as described below.

D. Parametric Survey

Figure (4) shows the threshold power, $P_{\rm thr}$ and orbit frequency, $\Omega_{\rm thr}$, as functions of beam waist radius, W_0 and sphere radius, a. These values are calculated through equations Eqs. (25). Stiffness coefficients k_r, k_ϕ are evaluated from Mie theory (section (Supplementary Note 2 B)), the mass assumes a density of Silica, $\rho = 6\pi\mu a$ and the drag coefficient $\xi = 6\pi\mu a$ makes use of the measured viscosity $\mu = 3.76 \times 10^{-6}$ Pa s, section (Supplementary Note 5). There are several points to note. First, threshold power and orbit frequencies become large as the sphere radius becomes small. This reflects the scaling properties of spin forces [7], which vanish in the case of a point dipole. Second, the threshold power varies most strongly with sphere radius. For the size of the sphere used in the experiment ($a = 0.77\mu$m), $P_{\rm thr}$ varies between ≈ 0.1 W and 1 W as the beam waist varies from $\approx 2\mu$m to $\approx 3.5\mu$m. Third, beyond a beam waist radius of $W_0 \approx 2\mu$m the orbit frequency, $\Omega_{\rm thr}$ is almost completely independent of W_0, and depends only weakly on a, for $a > 0.2\mu$m, expressing the fact that the ratio between spin and gradient forces is approximately constant for sufficiently large particles in sufficiently paraxial beams. As can be seen, the orbit frequency varies

between 10kHz and 20kHz for a variation in radius of $\pm 0.1\mu$m about the experimental value, $a = 0.77\mu$m, indicating the accuracy of the experiment in this regard. In passing, we note that it should be possible to generate very high orbit frequencies under easily obtained laboratory conditions. For a narrow beam waist ($\approx 0.5\mu$m), the threshold power, $P_{\rm thr}$, is ≈ 1 W for a sphere radius of $a \approx 0.18\mu$m. Under these conditions the orbit frequency $\Omega_{\rm thr} \approx 10^6$ Hz. The radius of such an orbit would be $\approx 0.25\mu$m.

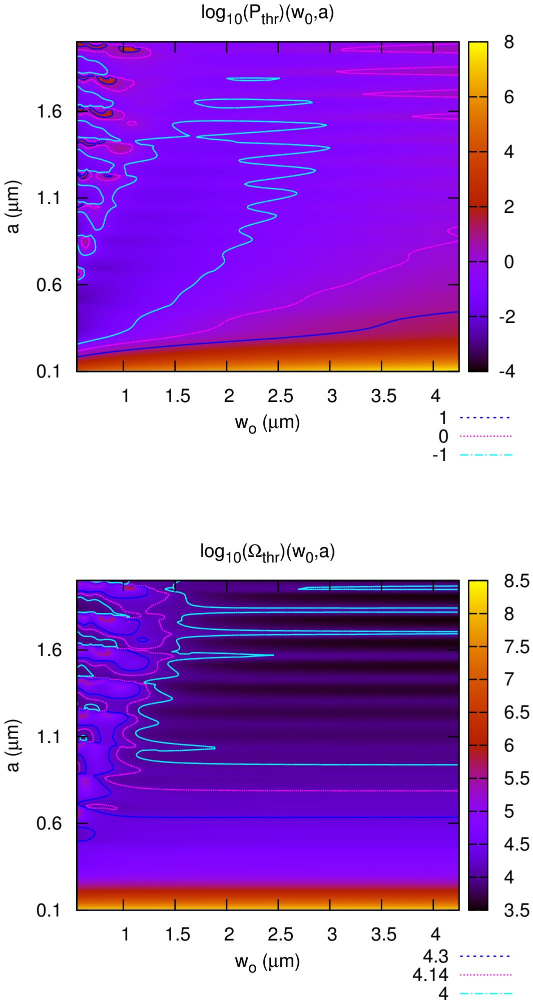

Supplementary Figure 4. Plots of base 10 logarithms of threshold power (a), $P_{\rm thr}$ (W), and orbit frequency (b) $\Omega_{\rm thr}$ (Hz) as functions of beam waist radius, W_0 and sphere radius, a. The contours of $\log_{10}(P_{\rm thr})$ are at $-1, 0, 1$ i.e. $P_{\rm thr} = 0.1$ W,1 W,10 W. The contours of $\log_{10}(\Omega_{\rm thr})$ coincide with $\Omega_{\rm thr} = 10$ kHz, 14.8 kHz, 20.0 kHz, with $\Omega_{\rm thr} = 14.8$ kHz the experimentally observed value.

Supplementary Note 4. ORBITAL MOTION

In the following we examine the orbital motion of particles in cicrularly polarized traps. Initially, we derive conditions for stable equilibria, ignoring thermal fluctuations. Next we quantify the stochastic fluctuations associated with the orbits.

A. Equilibrium Conditions

In the absence of thermal fluctuations, the equations of motion in polar coordinates are:

$$m(\ddot{r} - r\dot{\phi}^2) = Pf_r(r) - \xi\dot{r} \tag{44a}$$

$$m(2\dot{r}\dot{\phi} + r\ddot{\phi}) = Pf_\phi(r) - \xi r\dot{\phi} \tag{44b}$$

Where $f_{r,\phi}$ are the radial (i.e. gradient) and azimuthal spin forces, per unit power, P. Eq. (44a) is the radial component, Eq. (44b) tangential. These equations have a simple equilibrium solution, $r = 0$ which is also mechanically stable. However, above the threshold power, P_{thr}, introduced above, this solution is not thermally stable. Equilibrium orbits are formed when the azimuthal spin forces, Pf_ϕ balance the azimuthal drag, $\xi r\dot{\phi}$, and the gradient force Pf_r balances the centripetal force $mr\dot{\phi}^2$. Setting $r = r_\text{o}$, $\dot{\phi} = \Omega_\text{o}$ Eqs. (44), gives the equilibrium conditions,

$$\frac{P_\text{o}}{\xi^2} = -\frac{r_\text{o} f_r(r_\text{o})}{m f_\phi^2(r_\text{o})} \tag{45a}$$

$$\frac{\Omega_\text{o}}{\xi} = -\frac{f_r(r_\text{o})}{m f_\phi(r_\text{o})} \tag{45b}$$

P_o is the power required to sustain an orbit of radius r_o when the drag is ξ, and Ω_o is the associated frequency. Equilibrium conditions can be varied either through the power, P, or through the pressure in the cell, which determines the viscosity and therefore the drag, ξ. Since f_r/f_ϕ is approximately independent of radius, the orbit frequency is determined by this ratio, and by the drag, but does not depend explicitly on the optical power.

B. Linear Stability

The linear stability of the equilibrium orbits can be understood by first rewriting the equations of motion, Eq. (44) as coupled, first order equations,

$$\dot{r} = v \tag{46a}$$

$$m(\dot{v} - r\Omega^2) = Pf_r(r) - \xi v \tag{46b}$$

$$m(2v\Omega + r\dot{\Omega}) = Pf_\phi(r) - \xi r\Omega. \tag{46c}$$

Or,

$$\dot{r} = v \tag{47a}$$

$$\dot{v} = \frac{1}{m}\left[Pf_r(r) - \xi v + mr\Omega^2\right] \equiv \frac{1}{m}f_v(r, v, \Omega) \tag{47b}$$

$$\dot{\Omega} = \frac{1}{m}\left[\frac{1}{r}Pf_\phi(r) - \xi\Omega - \frac{2}{r}mv\Omega\right] \equiv \frac{1}{m}f_\Omega(r, v, \Omega) \tag{47c}$$

Put $r = r_\mathrm{o} + r_1(t)$, $v = v_1(t)$, $\Omega = \Omega_\mathrm{o} + \Omega_1(t)$. For small r_1, v_1, Ω_1:

$$\dot{r}_1 = v_1 \tag{48a}$$

$$\dot{v}_1 = r_1\frac{1}{m}\partial_r f_v\Big|_{\mathrm{eqm}} + v_1\frac{1}{m}\partial_v f_v\Big|_{\mathrm{eqm}} + \Omega_1\frac{1}{m}\partial_\Omega f_v\Big|_{\mathrm{eqm}} \tag{48b}$$

$$\dot{\Omega}_1 = r_1\frac{1}{m}\partial_r f_\Omega\Big|_{\mathrm{eqm}} + v_1\frac{1}{m}\partial_v f_\Omega\Big|_{\mathrm{eqm}} + \Omega_1\frac{1}{m}\partial_\Omega f_\Omega\Big|_{\mathrm{eqm}} \tag{48c}$$

Derivatives of f_v are :

$$\partial_r f_v\Big|_{\mathrm{eqm}} = P_\mathrm{o}(r_\mathrm{o})f_r'(r_\mathrm{o}) + m\Omega_\mathrm{o}^2 \tag{49a}$$

$$\partial_v f_v\Big|_{\mathrm{eqm}} = -\xi \tag{49b}$$

$$\partial_\Omega f_v\Big|_{\mathrm{eqm}} = 2mr_\mathrm{o}\Omega_\mathrm{o} \tag{49c}$$

Or, using Eqs. (45):

$$\partial_r f_v\Big|_{\mathrm{eqm}} = \frac{\xi^2}{m}\frac{f_r(r_\mathrm{o})}{f_\phi^2(r_\mathrm{o})}\left(f_r(r_\mathrm{o}) - r_\mathrm{o}f_r'(r_\mathrm{o})\right) \tag{50a}$$

$$\partial_v f_v\Big|_{\mathrm{eqm}} = -\xi \tag{50b}$$

$$\partial_\Omega f_v\Big|_{\mathrm{eqm}} = -2r_\mathrm{o}\xi\frac{f_r(r_\mathrm{o})}{f_\phi(r_\mathrm{o})} \tag{50c}$$

Derivatives of f_Ω are :

$$\partial_r f_\Omega\Big|_{\text{eqm}} = \frac{P_\text{o}(r_\text{o})\left(r_\text{o} f'_\phi(r_\text{o}) - f_\phi(r_\text{o})\right)}{r_\text{o}^2} \tag{51a}$$

$$\partial_v f_\Omega\Big|_{\text{eqm}} = -\frac{2m\Omega_\text{o}}{r_\text{o}} \tag{51b}$$

$$\partial_\Omega f_\Omega\Big|_{\text{eqm}} = -\xi \tag{51c}$$

Or, using Eqs. (45) :

$$\partial_r f_\Omega\Big|_{\text{eqm}} = \frac{1}{r_\text{o}}\frac{\xi^2}{m}\frac{f_r(r_\text{o})}{f_\phi^2(r_\text{o})}\left(f_\phi(r_\text{o}) - r_\text{o} f'_\phi(r_\text{o})\right) \tag{52a}$$

$$\partial_v f_\Omega\Big|_{\text{eqm}} = \frac{2\xi}{r_\text{o}}\frac{f_r(r_\text{o})}{f_\phi(r_\text{o})} \tag{52b}$$

$$\partial_\Omega f_\Omega\Big|_{\text{eqm}} = -\xi \tag{52c}$$

The system is asymptotically stable if and only if the real parts of all the eigenvalues of the matrix corresponding to Eqs. (48) are negative i.e. iff $\Re(\lambda_i) < 0 \quad \forall i$. Setting the determinant of Eq. (48) to zero gives the following polynomial, satisfied by the eigenvalues,

$$P(\lambda) = \lambda^3 + 2\frac{\xi}{m}\lambda^2 + \frac{\xi^2}{m^2}X\lambda + \frac{\xi^3}{m^3}Y. \tag{53}$$

With:

$$X(r_\text{o}) = 1 + 3\frac{f_r^2(r_\text{o})}{f_\phi^2(r_\text{o})} + r_\text{o}\frac{f_r(r_\text{o})f'_r(r_\text{o})}{f_\phi^2(r_\text{o})} \tag{54a}$$

$$Y(r_\text{o}) = \frac{f_r(r_\text{o})}{f_\phi^2(r_\text{o})}\left[f_r(r_\text{o}) + r_\text{o}\left(f'_r(r_\text{o}) - 2\frac{f_r(r_\text{o})}{f_\phi(r_\text{o})}f'_\phi(r_\text{o})\right)\right] \tag{54b}$$

$$\lambda = \frac{\xi}{m}l \tag{55}$$

Rescaling λ in Eq. (56)

$$P_l(l) = l^3 + 2l^2 + Xl + Y = 0. \tag{56}$$

The roots of $P_l(l) = 0$ determine the linear stability of the orbit. $P_l(l)$ depends only on the coefficients $X(r_\text{o})$ and $Y(r_\text{o})$, which depend only on the optical forces and are independent of the mass and the drag. Consequently, the orbital stability can be described solely in terms of orbit radius.

A complete stability analysis can be achieved by considering the discriminant of the cubic,

Eq. (56). This leads to rather complicated formulae. A simpler approach is provided below, resulting in formulae that are accurate for the system considered here.

1. The polynomial, $P_l(l) = 0$ has at least one purely real root which is negative, since $P_l(0) = Y > 0$ for all r_o. Call this l_0.

2. For small radii, r_o, the coefficients X, Y, Eq. (54) are approximately, $X \approx 1 + 4\frac{k_r^2}{k_\phi^2} \gg 1$, and $Y \approx 0$. Therefore, in addition to the negative real root, there are two further roots given by $l_\pm \approx -1 \pm \sqrt{1-X}$ which, since $X \gg 1$ are a complex conjugate pair with negative real part.

3. The real parts of the complex conjugate pair of roots, $l_\pm$ may change sign, destabilizing the orbits, for some greater radius. Demanding $\Re(l_\pm) = 0$ in Eq. (56) leads to the requirements that $[\Im(l_\pm)]^2 = X$, which arises when $2X(r_\mathrm{o}) = Y(r_\mathrm{o})$.

4. A necessary condition for there to exist three distinct, real roots is that $P_l(l)$ contains two turning points i.e. $P_l'(l)$ has two real roots, $l = -\frac{2}{3} \pm \frac{2}{3}\sqrt{1 - \frac{3}{4}X}$, requiring $X < \frac{4}{3}$.

The conditions in (3) and (4) can be estimated by assuming that the gradient and spin forces are proportional to the gradient of the beam intensity e.g.

$$f_r \approx -\gamma \frac{r}{W_0^2} \exp(-2r^2/W_0^2) \tag{57a}$$

$$f_\phi \approx -\frac{r}{W_0^2} \exp(-2r^2/W_0^2) \tag{57b}$$

Where W_0 is the beam waist radius and $\gamma \gg 1$ scales the radial force relative to the azimuthal force. This ignores the finite size of the particle, but compares favourably with numerical evaluations of the forces. Under this approximation, $X(r_\mathrm{o}) \approx 1 + 4\gamma^2(1 - \frac{r_\mathrm{o}^2}{W_0^2})$ and $Y(r_\mathrm{o}) \approx \gamma^2 4r_\mathrm{o}^2/W_0^2$. Condition (3) becomes,

$$2X(r_\mathrm{o}) = Y \Rightarrow \frac{r_\mathrm{o}^2}{W_0^2} \approx \frac{2}{3} + \frac{1}{6\gamma^2}, \tag{58}$$

so that orbits are asymptotically unstable for $r_\mathrm{o} \gtrsim \sqrt{2/3}W_0$. Condition (4) is,

$$X(r_\mathrm{o}) > \frac{4}{3} \Rightarrow \frac{r_\mathrm{o}^2}{W_0^2} > 1 - \frac{1}{12\gamma^2}, \tag{59}$$

so that orbits are completely unstable for $r_\mathrm{o} \gtrsim W_0$.

C. Orbits Fluctuations

When the optical power exceeds the threshold, $P_{\rm thr}$, mechanically stable orbits can form, as described above. For these orbits to be thermally stable and coherent, radial fluctuations must be small in comparison to the orbit radius, $r_{\rm o}$. These fluctuations can be estimated in a manner similar to sub-threshold fluctuations analysed in Section Supplementary Note 3. We consider perturbations to the equilibrium orbits in the presence of thermal fluctuations, and derive an expression for $\langle (r-r_{\rm o})^2\rangle$. Close to $P_{\rm thr}$, we find that $\langle (r-r_{\rm o})^2\rangle \propto 1/(P-P_{\rm thr})$: immediately above threshold fluctuations in the orbit radius are very large, and the orbit is incoherent despite being in mechanical equilibrium. This is related to the degree of linearity in the force field. A higher power, $P_{\rm orb}$ is required to propel the particle into non-linear regions where the orbits become coherent.

The Langevin equations of motion in polar coordinates are:

$$m\left(\ddot{r} - r\Omega^2\right) = Pf_r(r) - \xi\dot{r} + f_r^{\rm L} \tag{60a}$$

$$m\left(2\dot{r}\Omega + r\dot{\Omega}\right) = Pf_\phi(r) - \xi r\Omega + f_\phi^{\rm L} \tag{60b}$$

Where, as before, $f_{r,\phi}^{\rm L}$ are uncorrelated stochastic forces with zero mean and variance $2k_{\rm B}T\xi\delta(t-t')$. Perturbing the equilibrium orbit with $r \approx r_{\rm o}+r_1(t)$ and $\Omega \approx \Omega_{\rm o}+\Omega_1(t)$, and locally linearising the force field so that, for example $f_r(r_{\rm o}+r_1) \approx f_r(r_{\rm o}) + (r_1 - r_{\rm o})f_r'(r_{\rm o})$ and removing the lowest order (i.e. equilibrium) solution leaves, to first order:

$$m\left(\ddot{r}_1 - 2\Omega_{\rm o}r_{\rm o}\Omega_1 - \Omega_{\rm o}^2 r_1\right) = Pr_1 f_r'(r_{\rm o}) - \xi\dot{r}_1 + f_r^{\rm L} \tag{61a}$$

$$m\left(2\Omega_{\rm o}\dot{r}_1 + r_{\rm o}\dot{\Omega}_1\right) = Pr_1 f_\phi'(r_{\rm o}) - \xi r_{\rm o}\Omega_1 - \xi r_1\Omega_{\rm o} + f_\phi^{\rm L} \tag{61b}$$

The Fourier transform is:

$$m\left(-\omega^2\bar{r}_1 - 2\Omega_{\rm o}r_{\rm o}\bar{\Omega}_1 - \Omega_{\rm o}^2\bar{r}_1\right) = Pf_r'(r_{\rm o})\bar{r}_1 - i\omega\xi\bar{r}_1 + F_r^{\rm L} \tag{62a}$$

$$m(2i\omega\Omega_{\rm o}\bar{r}_1 + i\omega r_{\rm o}\bar{\Omega}_1) = Pf_\phi'(r_{\rm o})\bar{r}_1 - \xi r_{\rm o}\bar{\Omega}_1 - \xi\Omega_{\rm o}\bar{r}_1 + F_\phi^{\rm L} \tag{62b}$$

Where $\bar{r}_1, \bar{\Omega}_1$ are the FTs of r_1, Ω_1. This allows us to compute the power spectrum of the perturbed quantities, r_1 and Ω_1, in a manner directly analogous to the treatment in Section Supplementary Note 3 B. The result is:

$$\langle \mathbf{R}(\omega)\mathbf{R}^\star(\omega)\rangle = 2k_{\rm B}T\xi\mathbf{M}(\omega)\mathbf{M}^{\rm H}(\omega) \tag{63}$$

With,

$$\mathbf{M}^{(\mathrm{W})} = \frac{1}{r_{\mathrm{o}}\frac{\xi^3}{m}\left(-iW^3 - 2W^2 + iXW + Y\right)}\times$$
$$\begin{bmatrix} \xi(1+iW)r_{\mathrm{o}} & -2\xi\frac{f_r}{f_\phi}r_{\mathrm{o}} \\ -\frac{\xi^2}{m}\frac{f_r}{f_\phi}\left(-2iW + \left(\frac{r_{\mathrm{o}}f'_\phi}{f_\phi} - 1\right)\right) & -\frac{\xi^2}{m}\left(W^2 - iW - \frac{f_r^2}{f_\phi^2}\left(1 - \frac{r_{\mathrm{o}}f'_r}{f_r}\right)\right) \end{bmatrix} \tag{64}$$
$$\equiv \frac{1}{r_{\mathrm{o}}\frac{\xi^3}{m}P(W)}\begin{bmatrix} \xi m_{xx} & \xi m_{xy} \\ \frac{\xi^2}{m}m_{yx} & \frac{\xi^2}{m}m_{yy} \end{bmatrix}$$

Where the frequency has been rescaled according to $\omega = \frac{\xi}{m}W$. $P(W)$ is the polynomial, $P(W) = -iW^3 - 2W^2 + iXW + Y$ with coefficients:

$$X(r_{\mathrm{o}}) = 1 + \frac{f_r^2}{f_\phi^2}\left(3 + \frac{r_{\mathrm{o}}f'_r}{f_r}\right) \tag{65a}$$

$$Y(r_{\mathrm{o}}) = \frac{f_r^2}{f_\phi^2}\left(1 + r_{\mathrm{o}}\left(\frac{f'_r}{f_r} - 2\frac{f'_\phi}{f_\phi}\right)\right). \tag{65b}$$

The form of $P(W)$ is familiar from the analysis Section (Supplementary Note 3 A), for reasons associated with its construction.

As previously, time dependent variances may be computed through the inverse Fourier transform of the power spectrum. The instantaneous variance in the orbit radius is:

$$\langle r_1^2\rangle = \frac{2k_{\mathrm{B}}T}{r_{\mathrm{o}}^2}\frac{m}{\xi^2}\oint \frac{|m_{xx}(W)|^2 + |m_{xy}(W)|^2}{P^2(W)} \tag{66}$$

And,

$$\langle \Omega_1^2\rangle = \frac{2k_{\mathrm{B}}T}{r_{\mathrm{o}}^2}\frac{1}{m}\oint \frac{|m_{yx}(W)|^2 + |m_{yy}(W)|^2}{P^2(W)} \tag{67}$$

A basic property of the orbit fluctuations follows directly from the form of $P(W)$. For a linear force field with $f_r = k_r r$ and $f_\phi = k_\phi r$, the coefficient Y vanishes identically. When this happens, $W = 0$ is a characteristic frequency, and the variances $\langle r_1^2\rangle$ and $\langle \Omega_1^2\rangle$ diverge. For this reason, the forcefield must depart sufficiently from linearity in order for the orbit to be radially confined. Figure (5) shows a representative calculation of $\langle r_1^2\rangle$ as a function of optical power. We note that this approximation suffers from a number of inaccuracies (e.g. we have assumed that the force field is linear within the range of the fluctuations of the particle), and the results should not be treated as quantitatively meaningful. However, the qualitative message is clear. Orbits with small radius will not be coherent. In particular, the variance in the orbit radius is likely to take the particle into regions where the transverse

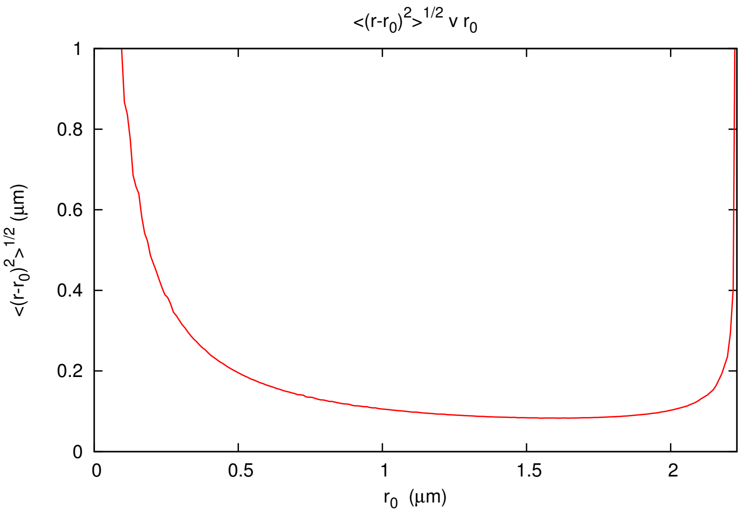

Supplementary Figure 5. Variance in orbit radius as a function of orbit radius

force is not only greatly reduced from the equilibrium value, but reversed completely. This process decreases the particle momentum resulting in the repeated formation and collapse of orbits. Only for sufficiently large orbit radius will the trajectory be stable and coherent. As a consequence, there is an intermediary regime above threshold power, $P_{\rm thr}$, in which the particle may acquire sufficient angular momentum to overcome gradient forces, but which may also suffer loss of momentum due to fluctuations. Therefore, a higher power, $P_{orb} > P_{\rm thr}$ is required to observe orbit formation. The hysteresis effect that we observe is associated with this.

Supplementary Note 5. DATA FITTING

The theoretical form of power spectral density (PSD, Eq. 29) for a particle in **regime I** can be rewritten using the following parameters:

$$p_1 = \frac{2k_{\rm B}T}{m} \tag{68}$$

$$p_2 = \frac{\xi}{m} \tag{69}$$

$$p_3 = \sqrt{\frac{K_r}{m}} = \omega_0 = \Omega_{\rm o} \tag{70}$$

$$p_4 = \sqrt{\frac{K_\phi}{m}} \tag{71}$$

$$\mathrm{PSD}_x = \frac{p_1 p_2[(p_3^2-\omega^2)^2+\omega^2 p_2^2+p_4^4]}{[(p_3^2-\omega^2)^2+\omega^2 p_2^2]^2+2p_4^4[(p_3^2-\omega^2)^2-\omega^2 p_2^2]+p_4^8} \tag{72}$$

This function tends to infinity at $p_4^2 = p_2 p_3$, which gives the limiting conditions in Eq. (5). The drag term p_2 does not change with power and thus we determined it from the fits to PSD in LP case ($p4 = 0$) with parallel polarization of both beams and took its mean value from x and y fits:

$$\mathrm{PSD}_x = \frac{p_1 p_2}{[(p_3^2-\omega^2)^2+\omega^2 p_2^2]^2} \tag{73}$$

We used the drag term as the fixed parameter in fits of theoretical PSDs to experimental ones. The system parameters were determined as follow in regime I

$$\omega_0 = \Omega_\mathrm{o} = p_3, \tag{74}$$

$$\frac{K_\phi}{K_r} \equiv \frac{f_\phi}{f_r} \equiv \frac{k_\phi}{k_r} = \frac{p_4^2}{p_3^2}. \tag{75}$$

In contrast in **regime II** we used Eq. (45b) to get

$$\frac{f_\phi}{f_r} = \frac{p_2}{p_3}. \tag{76}$$

Since the CMOS camera detects x and z coordinates only, we used the same calibration constant for both the x and y axes.

The mean radius of the particle trajectory was determined using a Gaussian probability distribution function in polar coordinates. We split the trajectory into 36 azimuthal sectors φ_i where we determined the parameters A_i, R_i, w_i of the following function:

$$f_{\varphi_i} = A_i r \exp\left[-\frac{(r-R_i)^2}{w_i^2}\right] \tag{77}$$

Finally the mean radius of the particle trajectory was determined as

$$\langle r\rangle = \frac{\sum_i r f_{\varphi_i}}{\sum_i f_{\varphi_i}} \tag{78}$$

The spin force f_ϕ was determined for regime I as

$$f_\phi = k_\phi \langle r\rangle = \frac{p_4^2 m\langle r\rangle}{P} \tag{79}$$

and for regime II we used Eq. (45a),

$$f_\phi = -\frac{\langle r\rangle \xi^2}{m}\frac{f_r}{f_\phi} = \frac{\langle r\rangle p_2 p_4 m}{P}, \tag{80}$$

where m is the mass of the particle. This fitting procedure was applied independently for each polarization setting and optical power, holding m and ξ fixed, and allowing all other parameters to vary. We find that the experimental measurements closely match theoretical values, with only a single scaling constant for the optical power. Crucially, the ratios for the radial and azimuthal forces (tabulated below), and their variation with radius (shown graphically in Fig. (1)) are almost identical. The need for rescaling the power arises from the variability of the experimental parameters. For instance, small uncertainties in the particle radius, density and refractive index strongly effect the absolute values of the force while the *ratios* of the radial and azimuthal forces are relatively insensitive to these variations.

Quantity	Experiment	Calculation
k_ϕ (N/Wm)	3.3×10^{-5}	1.58×10^{-5}
k_r (N/Wm)	2.38×10^{-4}	1.16×10^{-4}
$k_r/k_\phi \approx f_r(r)/f_\phi(r)$	0.138	0.14
Ω_{thr} (kHz)	14.8	14.9

[1] Beth, R. Mechanical detection and measurement of the angular momentum of light. *Phys. Rev.* **50**, 115–125 (1936).

[2] Barnett, S. M. *et al.* On the natures of the spin and orbital parts of optical angular momentum. *J. Opt.* **18**, 064004 (2016).

[3] Pfeifer, R. N. C., Nieminen, T. A., Heckenberg, N. R. & Rubinsztein-Dunlop, H. Colloquium: Momentum of an electromagnetic wave in dielectric media. *Rev. Mod. Phys.* **79**, 1197–2016 (2007).

[4] Bliokh, K. Y., Bekshaev, A. Y. & Nori, F. Optical momentum and angular momentum in complex media: from the Abraham-Minkowski debate to unusual properties of surface plasmon-polaritons. *New. J. Phys.* **19**, 123014 (2017).

[5] Berry, M. V. Optical currents. *J. Opt. A* **11**, 094001 (2009).

[6] Bliokh, K. Y., Bekshaev, A. Y. & Nori, F. Dual electromagnetism: helicity, spin, momentum, and angular momentum. *New. J. Phys.* **15**, 033026 (2013).

[7] Bliokh, K. Y., Bekshaev, A. Y. & Nori, F. Extraordinary momentum and spin in evanescent waves. *Nat. Commun.* **5**, 3300 (2014).

[8] Antognozzi, M. *et al.* Direct measurements of the extraordinary optical momentum and transverse spin-dependent force using a nano-cantilever. *Nat. Phys.* **12**, 731–735 (2016).

[9] Liu, L. *et al.* Three-dimensional measurement of the helicity-dependent forces on a Mie particle. *Phys. Rev. Lett.* **120**, 223901 (2018).

[10] Bekshaev, A., Bliokh, K. Y. & Soskin, M. Internal flows and energy circulation in light beams. *J. Opt.* **13**, 053001 (2011).

[11] Jackson, J. *Classical Electrodynamics: Third Edition* (Cambridge university press, 1999).

[12] Marques, M. I. & Jose Saenz, J. Scattering forces and electromagnetic momentum density in crossed circularly polarized standing waves. *Opt. Lett.* **37**, 2787–2789 (2012).

[13] Coffey, W., Kalmykov, Y. P. & JT, W. *The Langevin Equation* (World Scientific, 1996).

[14] Beresnev, S., Chernyak, V. & Fomyagin, G. Motion of a spherical-particle in a rarefied gas. 2. drag and thermal polarization. *J. FLuid Mech.* **219**, 405–421 (1990).
